# Internet Access and Usage Among Stroke Survivors and Their Informal Caregivers: Cross-sectional Study

**DOI:** 10.2196/25123

**Published:** 2021-03-08

**Authors:** Imama Ali Naqvi, Tahani Casameni Montiel, Yazan Bittar, Norma Hunter, Munachi Okpala, Constance Johnson, Mark G Weiner, Sean Savitz, Anjail Sharrief, Jennifer Elizabeth Sanner Beauchamp

**Affiliations:** 1 Division of Stroke and Cerebrovascular Diseases Department of Neurology Columbia University Irving Medical Center New York, NY United States; 2 Department of Nursing Research Cizik School of Nursing The University of Texas Health Science Center at Houston Houston, TX United States; 3 Lewis Katz School of Medicine Temple University Philadelphia, PA United States; 4 Department of Neurology Institute for Stroke and Cerebrovascular Disease The University of Texas Health Science Center at Houston Houston, TX United States; 5 Department of Neurology Institute for Stroke and Cerebrovascular Disease McGovern Medical School, The University of Texas Health Science Center at Houston Houston, TX United States; 6 Department of Nursing Research Cizik School of Nursing and School of Bioinformatics The University of Texas Health Science Center at Houston Houston, TX United States; 7 Department of Population Health Sciences Weill Cornell Medicine New York, NY United States; 8 Department of Nursing Research Cizik School of Nursing, Institute for Stroke and Cerebrovascular Disease The University of Texas Health Science Center at Houston Houston, TX United States

**Keywords:** internet access, stroke, caregivers, surveys, questionnaires, mobile phone

## Abstract

**Background:**

Web-based interventions have shown promise for chronic disease management but have not been widely applied to populations with stroke. Existing barriers may inhibit the adoption of web-based interventions among stroke survivors and necessitate the involvement of informal caregivers. However, limited information is available on internet accessibility and usability among stroke survivors and their caregivers.

**Objective:**

This study aims to investigate internet access and usage in a cohort of stroke survivors and their caregivers.

**Methods:**

A cross-sectional survey was conducted with 375 participants (248 stroke survivors and 127 caregivers). Descriptive statistics were generated using cross-tabulation. Comparisons with categorical data were conducted using the chi-square test, whereas the Mann-Whitney *U* test was used for comparisons involving ordinal variables.

**Results:**

Overall, 86.1% (323/375) of the participants reported having internet access. Caregivers were more likely than stroke survivors to access the internet (N=375, χ^2^_1_=18.5, *P*<.001) and used text messaging (n=321, χ^2^_1_=14.7, *P*<.001). Stroke survivors and caregivers with internet access were younger than stroke survivors and caregivers without internet access. The highest number of participants who reported internet access were non-Hispanic White. Smartphones were the most common devices used to access the internet. Email was the most common type of internet usage reported. Patients who survived for >12 months after a stroke reported higher internet access than those who survived <3 months (*P*<.001). The number of hours per week spent using the internet was higher for caregivers than for stroke survivors (*P*<.001).

**Conclusions:**

Future feasibility and acceptability studies should consider the role of the informal caregiver, participant age, race and ethnicity, the use of smartphone apps, email and text correspondence, and the amount of time elapsed since the stroke event in the design and implementation of web-based interventions for populations with stroke.

## Introduction

### Background

In the United States, 795,000 people experience stroke annually [1]. Although improvements in the acute management of stroke have led to a decline in associated mortality, stroke-related morbidity leads to chronic disability in approximately half of all stroke survivors [2]. Comprehensive poststroke interventions should consider common stroke sequelae, including functional disabilities (eg, limb paralysis and sensory disturbances), speech disabilities (eg, aphasia), emotional disturbances (eg, poststroke anxiety and depression), and cognitive impairments (eg, impaired memory) [1]. Mobility is reduced in more than half of all stroke survivors aged ≥65 years [3]. Difficulty in producing and understanding speech, known as aphasia, occurs in an estimated 25%-40% of stroke survivors [4]. The prevalence of poststroke anxiety and depression ranges from 21% to 29% and from 29% to 31%, respectively [5-7]. Stroke is the second most common cause of cognitive impairment and dementia, with approximately 30% of stroke survivors experiencing cognitive impairment [8] or dementia [9]. Despite the complex needs of stroke survivors, poststroke care systems are inadequate. Stroke survivors may have limited access to outpatient care because of impaired mobility, limited access to transportation, and lack of support [10]. Most stroke survivors are discharged home from the hospital and receive care provided primarily by unprepared informal caregivers (eg, spouses and family members) [11,12]. Measures that expand access to poststroke care and comprehensively address the challenges that stroke survivors and their caregivers encounter are needed [13].

Web-based telehealth interventions have been found to be effective for acute stroke care, potentially beneficial for extended neurology care in rural areas [14], and cost-effective in stroke and dementia populations [15,16].

### Purpose

Although a number of studies have examined internet access and usage among populations other than those with stroke (eg, patients with diabetes) [17], studies are needed to determine the feasibility and acceptability of web-based interventions across diverse stroke populations with complex disabilities and various levels of ability [18]. Uncertainties remain regarding the implementation of web-based interventions, including suitable stroke survivors and necessary stroke survivor support structures [19], as well as the role of the caregiver. Only 67% of US adults aged ≥65 years reported internet access compared with 44% of adults aged ≥80 years [20]. The lower rates of internet access with advancing age present an additional challenge, as an estimated three-fourths of all strokes occur in adults aged ≥65 years [21] and 17% of all strokes occur in adults aged >85 years [22]. Non-Hispanic Black and Hispanic US adults are less likely than non-Hispanic White adults to have access to the internet in their home environment [23]. The highest increase in stroke prevalence (29%) is estimated to be reported among Hispanic men [1], and known racial and ethnic disparities occur in almost every aspect of stroke care [24]. To develop appropriate and accessible web-based interventions for stroke survivors and caregivers, we must fully understand these digital disparities within the context of stroke survivorship. Therefore, the purpose of our study was to investigate internet access and usage in a cohort of stroke survivors and their caregivers.

## Methods

### Design and Sampling

This was an observational study of cross-sectional survey data collected from a convenience sample of stroke survivors and their caregivers from 2 large metropolitan areas: Houston, Texas, and Philadelphia, Pennsylvania. Institutional review board (IRB) approval and authorization for data sharing were obtained from both participating universities. As the only record linking the participant and the research was the informed consent document, the IRBs waived the requirement for written informed consent. Each participant received a letter of information outlining the study, and completion of the survey was taken as the form of consent to participate.

### Recruitment

Recruitment sites included outpatient clinics, inpatient units, and community support groups. Participants were recruited during a routine visit to an outpatient stroke clinic within the Texas Medical Center (Houston, TX). The outpatient stroke clinic manages care of racially and ethnically diverse stroke survivors and treated approximately 1000 new stroke survivors in 2019. Participants were also recruited at an annual Houston community stroke festival and 2 Houston-area stroke support groups to supplement recruitment. In Philadelphia, Pennsylvania, participants were recruited from a comprehensive stroke center (CSC) and affiliated outpatient stroke clinic. The CSC serves a diverse minority and medically underserved population and treated approximately 600 patients with acute stroke in 2019. Screening measures to determine eligibility criteria included accessing electronic health records and participant self-reports. Identifiers (eg, names) collected for approaching potential participants were not retained.

Eligibility criteria for patients from both participating universities were as follows: patients who (1) were aged ≥18 years, (2) spoke and read English or Spanish, and (3) had a history of stroke or self-identified as an informal caregiver of a stroke survivor. In Houston, surveys were collected in person using an Apple iPad and Research Electronic Data Capture (REDCap) [25,26] survey links or paper surveys and manually entered into REDCap [25,26] by a trained research member. Surveys with the survey links were also emailed or completed by telephonic interviews by a trained research member who manually entered the data into REDCap [25,26]. Surveys were carried out from September 2018 to July 2019. In Philadelphia, in-person surveys were collected directly via REDCap [25,26] survey links on an iPad. Surveys were carried out from March 2019 to July 2019. All surveys were assigned a study identifier without personal identifiers. The mode of data collection was dependent on user comfort and iPad availability. All data were collected when trained surveyors were available, except for the Houston site, which also emailed REDCap [25,26] survey links for participants to complete. REDCap [25,26] is a secure, web-based app that supports data capture and export procedures at both universities.

### Variables

The survey was not intended to collect psychometric data and thus did not rely on a validated psychometric instrument. However, contributions from experts in neurology, nursing, and bioinformatics were used to create a 14-question survey in English and Spanish languages (Multimedia Appendix 1). In total, 8 demographic questions included variables such as gender, race, ethnicity, and health insurance status. The duration in months from stroke events for stroke survivors and relationship (eg, spouse) between stroke survivors and caregivers were obtained. A total of 6 internet access and usage questions included an inquiry into the form of internet access at home, including cellular phone data as well as types of devices used to access the internet. Modes of communication, including email, text messages, web browsing, and gaming interactions, were included as discrete queries. Time spent and the language predominately used while on a device used to access the internet were separated for choice.

### Statistical Analysis

Data were analyzed using SPSS 25.0. Participants who selected being both stroke survivors and caregivers were counted as stroke survivors for the analysis. Descriptive data were generated to explore trends in internet access and usage through cross-tabulation. Comparisons with categorical data were done using the chi-square test, whereas the Mann-Whitney U test was used for comparisons involving ordinal variables. On the basis of a sample size of 127, a one-sample chi-square test had 80% power when the hypothesized proportion of internet users was 0.9, and the proportion of internet users in the sampled population was 0.8 [27]. As we planned to test this hypothesis in both stroke survivors and caregivers, we recruited 248 stroke survivors and 127 caregivers.

## Results

### Sample Characteristics of Stroke Survivors and Informal Caregivers

Of the 397 surveys collected, 375 were analyzed (Table 1). Overall, 22 surveys were excluded because 19 participants did not indicate stroke survivors or caregiver status, and 3 participants did not complete the internet access and usage questions. Of the 375 surveys, 248 (66.1%) were from stroke survivors and 127 (33.9%) were from caregivers. Overall, 45.3% (169/373) of the participants were male, and 89.4% (329/368) reported having health insurance. Most participants (54/127, 42.5%) reported a spousal caregiver relationship to the care recipient, followed by a child caregiver relationship (39/127, 30.7%) to the parent care recipient. Participants (318/375, 84.8%) were primarily recruited from outpatient sites. A total of 107 of the 375 (28.5%) participants surveyed were from Philadelphia sites, including 58 (54.2%) from inpatient units and 49 (45.8%) from an outpatient clinic.

**Table 1:** Comparison of demographic characteristics of stroke survivors and informal caregivers (N=375) by internet access.

Characteristics		Total participants^a^	With internet access^b^ (n=323, 86.1%)		Without internet access^b^ (n=52, 14%)	
			Informal caregivers (n=123)	Stroke survivors (n=200)	Informal caregivers (n=4)	Stroke survivors (n=48)
Age (years), mean (SD)		58	51 (14)	59 (14)	60 (19)	69 (12)
Sex (male), n (%)		169 (45.3)	33 (26.8)	108 (54.3)	1 (25)	27 (57.4)
Health insurance, n (%)		329 (89.4)	100 (82)	185 (94.9)	4 (100)	40 (85)
**Race and ethnicity, n (%)**						
	Non-Hispanic Black	132 (35.3)	35 (29)	71 (36)	1 (33)	25 (52)
	Non-Hispanic White	114 (30.4)	48 (39)	63 (32)	—^c^	3 (6)
	Hispanic	91 (24)	31 (25)	44 (22)	2 (67)	14 (29)
	Other	37 (10)	9 (7)	22 (11)	—	6 (13)
**Time since stroke (months), n (%)**						
	<3	73 (30)	—	48 (24)	—	25 (57)
	3-6	33 (14)	—	28 (14)	—	5 (11)
	6-12	33 (14)	—	29 (15)	—	4 (9)
	>12	102 (42.3)	—	92 (47)	—	10 (23)
**Relationship^d^, n (%)**						
	Parent	11 (9)	9 (7)	—	2 (50)	—
	Spouse	54 (43)	52 (42)	—	2 (50)	—
	Son or daughter	39 (31)	39 (32)	—	—	—
	Sibling	6 (5)	6 (5)	—	—	—
	Grandchild	4 (3)	4 (3)	—	—	—
	Friend	13 (11)	13 (11)	—	—	—

^a^Values in this column represent the number of participants who answered the specific demographic survey item.

^b^Counts may not add up to the n value indicated in the column header because of missing data. Percentages may not add up to 100% because of rounding off.

^c^Not available.

^d^Caregiver’s relationship with the care recipient.

Stroke survivors with internet access were younger (mean 59, SD 14 years) than stroke survivors without internet access (mean 69, SD 12 years). Similarly, caregivers with internet access were younger (51, SD 14 years) than those without internet access (mean 60, SD 19 years). Overall, 36.4% (71/197) non-Hispanic Black, 31.9% (63/197) non-Hispanic White, and 22.3% (44/197) Hispanic stroke survivors reported internet access. Fifty two percent (25/48) non-Hispanic Black, 6.2% (3/48) non-Hispanic White, and 29.1% (14/48) Hispanic stroke survivors reported no internet access. Of those that reported internet access, 28.5% (35/123) non-Hispanic Black, 39% (48/123) non-Hispanic White, and 25.2% (31/123) Hispanic caregivers reported internet access. One (1/3, 33%) non-Hispanic Black and two (2/3, 67%) Hispanic caregivers reported no internet access. More stroke survivors with stroke events >12 months ago (n=197) had internet access as compared with those with stroke events <3 months ago (n=44). The difference in internet access between these 2 groups of stroke survivors was statistically significant (*U*=2782.0, *z*=−3.93, *P*<.001).

### Characteristics of Stroke Survivor and Informal Caregiver Internet Users

Overall, 86.1% (323/375) of participants reported internet access (Table 2). Compared with an estimate that 89% of American adults have internet access [27,28], 80.6% (200/248) of stroke survivors (*P*<.001) and 96.8% (123/127) of caregivers (*P*=.001) had access. Caregivers were more likely than stroke survivors to access the internet (N=375, χ^2^_1_=18.5, *P*<.001). Smartphones were the most common type of device used to access the internet. Of the stroke survivors, 82.5% (165/200) reported using a smartphone, 59.5% (119/200) reported using a computer, and 40.5% (81/200) reported using an iPad to access the internet. Similarly, 91.1% (112/123) caregivers reported using a smartphone, 78% (96/123) reported using a computer, and 57.7% (71/123) reported using an iPad to access the internet. Email was the most common type of internet usage reported among stroke survivors (143/200, 71.2%) and caregivers (110/123, 89.4%), followed by browsing the web (stroke survivors=122/200, 61%; caregivers=104/123, 84.6%) and video games (stroke survivors=40/200, 20%; caregivers=42/123, 34.1%). The number of hours per week spent using the internet by caregivers (n=122) was higher than that of stroke survivors (n=194), and the difference was statistically significant (*U*=8922.00, *z*=−3.81, *P*<.001). The majority of stroke survivors (177/200, 88.5%) and caregivers (116/123, 94.3%) reported English as the primary language used in their devices. Caregivers were more likely to use text messaging than stroke survivors (n=321, χ^2^_1_=14.74, *P*<.001).

**Table 2:** Internet usage characteristics of stroke survivors and informal caregivers.

Internet usage characteristics			Total participants with internet access^a^ (n=323)	
			Informal caregivers (n=123)	Stroke survivors (n=200)
**Type of device, n (%)**				
	Smartphone		112 (91.1)	165 (82.5)
	Computer		96 (78)	119 (59.5)
	iPad or tablet		71 (58)	81 (40.5)
	Other		8 (7)	8 (4)
	Do not access the internet^b^		—^c^	4 (2)
**Internet usage, n (%)**				
	Email		110 (89.4)	143 (71.2)
	Web pages		104 (84.6)	122 (61)
	Video games		42 (34)	40 (20)
	Other		30 (24)	40 (20)
	Do not access the internet^b^		1 (1)	23 (12)
**Internet hours per week (hours), n (%)**				
	0-5		27 (22)	87 (45)
	6-10		28 (23)	38 (20)
	11-15		25 (20)	25 (13)
	16-20		14 (12)	13 (7)
	21-25		6 (5)	5 (3)
	>25		22 (18)	26 (13)
**Device language used, n (%)**				
	English		116 (94.3)	177 (88.5)
	Spanish		14 (11)	25 (12.5)
	Other		4 (3)	6 (3)
**Text messaging**				
	Yes, n (%)		104 (84.6)	121 (60.5)

^a^Counts may not add up to the n values indicated in the column header because of missing data. Percentages may not add up to 100% because of rounding off.

^b^Home internet access is available but does not personally access the internet from home.

^c^Not available.

## Discussion

### Principal Findings

Overall, most of the sampled stroke population reported having internet access. Compared with a national estimate of adults with internet access, fewer stroke survivors reported internet access, whereas a greater number of caregivers reported access. Caregivers were more likely to access the internet and spend more time per week using the internet than stroke survivors. Stroke survivors and caregivers with internet access were younger than those without internet access. Internet access was significantly higher in stroke survivors more than 12 months after stroke than in stroke survivors less than 3 months after stroke. Smartphones were the most common devices used to access the internet.

### Comparison With Prior Work

Overall, 85% (323/375) of the participants reported internet access, which is lower than that of the general public [27,29], but higher than the 72% for US adult internet users living within the confines of a chronic condition [30]. Similar to our findings, mobile devices and computers are commonly used platforms [31] for accessing the internet and may be feasible and acceptable platforms for providing web-based stroke recovery interventions. Optimizing web-based interventions to be accessible by smartphones and computers may increase accessibility, given the predominance of smartphone and computer access among this sample. Lesser internet technology use among aging patients and their caregivers compared with younger adults [30] may indicate that these platforms may be challenging for the older stroke population. However, stroke is no longer a chronic condition in older individuals alone. The increasing rates (men 41.5% and women 30%, aged 35-44 years) of acute ischemic stroke from 2003 to 2012 in young adults coexist with the increasing prevalence of traditional risk factors [32,33] and emphasize the importance of focusing on web-based stroke recovery efforts in younger adults. Ischemic stroke events have increased significantly in adults aged 18-54 years [32]. Furthermore, the perception of the *digital divide* based on advancing age is rapidly changing, as internet use becomes more pervasive in the United States. Adults in the United States are reporting internet usage during the COVID-19 pandemic, with 84% of individuals aged ≥50 years, 98% of individuals aged 30-49 years, and 100% of individuals aged 18-29 years of age reporting internet usage or owning a smartphone device [34]. It is likely that web-based approaches will become more feasible and acceptable with the changing exposures and needs of technologically diverse stroke populations.

Chronic stroke survivors (stroke event>12 months ago) reported the highest rates of internet access, whereas those less than 3 months poststroke had the least access. This suggests that access and ease of internet use may be more robust in chronic poststroke care. Web-based stroke recovery interventions may require personalized strategies for stroke survivors’ recovery status and individual preferences [15]. Stroke recovery care may also be limited by physical and geographical barriers. Although not among stroke survivors, web-based telehealth visits have been successfully used in the general population and have been shown to improve blood pressure control in hypertensive patients [35]. These web-based visits may be used as a platform to capture poststroke patient-reported outcomes and implement interventions centered on secondary stroke prevention, such as risk factor control, medication adherence, and lifestyle modifications. The COVID-19 pandemic has highlighted the utility of web-based telehealth visits. For example, the rapid transition to telehealth visits for outpatient care among patients with neurological diseases has been implemented, allowing patients to communicate with health care providers via smartphones and other devices [36]. As web-based telehealth services expand, health care professionals are likely to learn more about the feasibility and accessibility of these web-based services across different populations with incentivization to assemble infrastructures for effective implementation of web-based poststroke care.

Because most stroke survivors are discharged home from the hospital, many only receive care from unprepared caregivers [37]. The amount of care provided by the caregiver to the stroke survivor appears to increase significantly immediately after hospital discharge and remains high throughout the first 12 months after the stroke [38]. Web-based interventions that actively engage caregivers may improve postacute stroke care. Caregivers were more likely to access the internet and spend more time using on it than stroke survivors. Caregivers of individuals with chronic conditions appear to use the internet for general purposes, to access health-related information and track health-related indicators (eg, weight), support, and services [30,31]. Caring for individuals with a chronic condition is considered a major life stressor that negatively affects the health and well-being of caregivers [39-42]. However, stroke survivor-informal caregiver dyad interventions have predominately focused on the health and well-being of stroke survivors rather than caring for oneself as a caregiver [40]. Although more studies are needed to determine the effectiveness of web-based interventions aimed at meeting the needs of caregivers [40,43], the American Heart Association or American Stroke Association recommends web-based stroke recovery interventions that meet the evolving needs of technologically advanced caregivers of stroke survivors [40]. Optimal stroke recovery requires web-based strategies that target the health and well-being of both stroke survivors and caregivers, with both being active participants [40].

The highest number of participants who reported internet access were non-Hispanic Whites. Racial differences in internet access and technology usage emphasize the need to address known disparities [28]. Racial and ethnic disparities also exist in stroke recovery care. Non-Hispanic Black and Hispanic participants receive less intensive stroke rehabilitation, education, and counseling than non-Hispanic White participants [44,45]. Minority groups, including non-Hispanic Black and Hispanic participants, have a higher risk of stroke [3]. The highest increase in stroke prevalence was observed in Hispanic men [3]. Non-Hispanic Black participants have a higher prevalence of uncontrolled blood pressure, which is the most important risk factor for stroke [46]. Notably, efforts to reduce racial and ethnic disparities in blood pressure control among stroke survivors have not been effective [47]. Web-based interventions to reduce stroke recurrence and improve risk factor control could address this gap in care by providing internet services and devices to individuals from the highest risk groups. Considerations for language barriers should also be given in web-based poststroke care.

### Limitations

One study limitation is that the sample may not be representative of all US stroke survivors and their informal caregivers. Therefore, the results should be interpreted with caution.

Detailed information regarding socioeconomic status and urban or rural location was not obtained. Future studies of internet access and use should consider targeted oversampling of economically disadvantaged stroke populations and stroke survivors and caregivers living in remote areas with limited broadband connectivity. Socioeconomic details can be gauged by collecting individual- and neighborhood-level social determinants of health data. Geo-mapping using ZIP codes for areas with the highest socioeconomic disparities and geographic barriers to classify the type and range of web-based services rendered will be useful before system-wide implementation of web-based stroke recovery interventions. The sample size was adequate, and the sample was racially and ethnically diverse; however, the nonprobability convenience sampling strategy may have resulted in selection bias. Stroke survivors and caregivers not surveyed were likely missed at random because efforts were limited to surveyor availability. Furthermore, the generalizability of the results is limited by the lack of nonparticipant data (eg, demographics and reasons). However, the sample came from 2 large urban areas providing data from considerably underinvestigated minority stroke survivor and caregiver populations. Although the survey was developed and edited by a multidisciplinary team, the internal consistency and content validity of the survey were not tested.

### Conclusions

Web-based interventions following stroke should consider the role of the caregiver, participant age, race, ethnicity, the use of smartphone apps, email and text correspondence, and the amount of time since the stroke event. The results suggest that web-based interventions may be feasible and acceptable for certain stroke survivors and caregivers. Future feasibility and acceptability studies should consider these findings when designing and implementing web-based stroke recovery interventions to minimize barriers to access, tailor the intervention to maximize adherence, and target those most likely to use web-based resources.

## Appendix

Multimedia Appendix 1 Internet Usage Survey Questions.

## Abbreviations

CSC: comprehensive stroke center
IRB: institutional review board
REDCap: Research Electronic Data Capture
