# Supplementary material for: Internet Access and Usage Among Stroke Survivors and Their Informal Caregivers: Cross-sectional Study
Source: JMIR Form Res. 2021 Mar 8;5(3):e25123. doi: 10.2196/25123 (PMC7985796; doi:10.2196/25123)
Supplement: Multimedia Appendix 1 [file formative_v5i3e25123_app1.docx]

| **Survey Questions- Spanish** | |
| --- | --- |
|  | 1. ¿Cuál es su edad en años? |
|  | 2. ¿Cuál es su género? |
|  | 3. ¿Usted tiene seguro de salud? |
|  | 4. ¿Con qué raza se identifica usted? |
|  | 5. ¿Que considera que es su origen étnico? |
|  | 6. ¿Es usted un sobreviviente de un derrame cerebral? |
|  | 7. Si es usted un sobreviviente de un derrame cerebral, ¿hace cuánto sucedió su más reciente derrame cerebral? |
|  | 8. ¿Es usted un cuidador, (por ejemplo, un padre, esposo, hijo, hermano, amigo) de alguien que tuvo un derrame cerebral? |
|  | 9. ¿Usted tiene algún tipo de acceso al internet en casa, incluyendo celulares? |
|  | 10. ¿Qué tipo de dispositivo(s) electrónico(s) utiliza en su hogar para acceder al internet? |
|  | 11. ¿Para que utiliza el internet en casa? |
|  | 12. ¿Cuantas horas cada semana usted usa el internet por cualquier razón (por ejemplo, correo electronico, jugar juegos de video, buscando el internet, leer libros o periódicos, etc.)? |
|  | 13. ¿Qué idioma usa principalmente en sus dispositivos electrónicos en casa? |
|  | 14. ¿Usted recibe y envía mensajes de texto? |

**Supplementary Table 1**. Internet Usage Survey Questions- Spanish Version

**Supplementary Table 2**. Internet Usage Survey Questions- English Version

| **Survey Questions** | |
| --- | --- |
|  | 1. What is your age in years? |
|  | 2. What is your gender? |
|  | 3. Do you have health insurance? |
|  | 4. What race do you identify as? |
|  | 5. What do you consider your ethnicity to be? |
|  | 6. Are you a stroke survivor? |
|  | 7. If you are a stroke survivor, how long ago was your most recent stroke? |
|  | 8. Are you a caregiver (for example, parent, spouse, child, sibling, friend) of someone who had a stroke? |
|  | 9. Do you have any form of access to the Internet at home, including cellular phone data? |
|  | 10. What type(s) of electronic devices do you use at home to access the Internet? |
|  | 11. What do you use the Internet for at home? |
|  | 12. How many hours each week do you use the internet at home for any reason (for example, emailing, playing video games, searching the internet, reading books or newspaper, etc.)? |
|  | 13. What language do you mostly use on your electronic device at home? |
|  | 14. Do you receive and send text messages? |
